# Dissecting Adaptive Traits with Nested Association Mapping: Genetic Architecture of Inflorescence Morphology in Sorghum

**DOI:** 10.1534/g3.119.400658

**Published:** 2020-03-26

**Authors:** Marcus O. Olatoye, Sandeep R. Marla, Zhenbin Hu, Sophie Bouchet, Ramasamy Perumal, Geoffrey P. Morris

**Affiliations:** * Department of Agronomy, Kansas State University, Manhattan, Kansas 66506; † Kansas State University, Agricultural Research Center, Hays, Kansas 67601

**Keywords:** Adaptation, Climate, Crops, Genome-wide association studies, Plant development, MPP, Multiparental Populations

## Abstract

In the cereal crop sorghum (*Sorghum bicolor*) inflorescence morphology variation underlies yield variation and confers adaptation across precipitation gradients, but its genetic basis is poorly understood. We characterized the genetic architecture of sorghum inflorescence morphology using a global nested association mapping (NAM) population (2200 recombinant inbred lines) and 198,000 phenotypic observations from multi-environment trials for four inflorescence morphology traits (upper branch length, lower branch length, rachis length, and rachis diameter). Trait correlations suggest that lower and upper branch length are under somewhat independent control, while lower branch length and rachis diameter are highly pleiotropic. Joint linkage and genome-wide association mapping revealed an oligogenic architecture with 1–22 QTL per trait, each explaining 0.1–5.0% of variation across the entire NAM population. There is a significant enrichment (2.twofold) of QTL colocalizing with grass inflorescence gene homologs, notably with orthologs of maize *Ramosa2* and rice *Aberrant Panicle Organization1* and *TAWAWA1*. Still, many QTL do not colocalize with inflorescence gene homologs. In global georeferenced germplasm, allelic variation at the major inflorescence QTL is geographically patterned but only weakly associated with the gradient of annual precipitation. Comparison of NAM with diversity panel association suggests that naive association models may capture some true associations not identified by mixed linear models. Overall, the findings suggest that global inflorescence diversity in sorghum is largely controlled by oligogenic, epistatic, and pleiotropic variation in ancestral regulatory networks. The findings also provide a basis for genomics-enabled breeding of locally-adapted inflorescence morphology.

Understanding the genetic architecture of complex traits in crops provides insights into crop evolution and guidance on breeding strategies. Adaptive traits are phenotypic characteristics that are subject to selection toward an optimum for a particular environment (Barrett and Hoekstra 2011). Genetic architecture describes the structure of the genotype-phenotype map for complex traits in populations: the number of loci, distribution of effect size, frequencies of alleles, gene action (dominance and epistasis), and the degree of linkage and pleiotropy (Holland 2007). A complex trait may be oligogenic or polygenic, depending on whether few or many loci contribute to the trait variation, respectively (Timpson *et al.*, 2018). Trait variation in a population may shift from oligogenic to polygenic architecture as a population moves toward an optimum (*i.e.*, Fisher-Orr model) (Orr 2005; Tenaillon 2014). Thus, characterizing genetic architecture of complex traits under natural and/or artificial selection is a key step to bridge theoretical understanding (*e.g.*, evolutionary, metabolic, or developmental drivers) and applied outcomes (*e.g.*, crop and livestock breeding strategies, or management of human genetic disorders) (Hansen 2006; Timpson *et al.* 2018). For instance, molecular breeding strategies are guided by genetic architecture, with marker-assisted backcross for monogenic traits, marker-assisted recurrent selection for oligogenic traits, and genomic selection for polygenic traits (Bernardo 2008).

Divergence of adaptive traits often results in genetic differentiation and population structure that hinders effective characterization of their genetic architecture (Myles *et al.* 2009; Brachi *et al.* 2011). Genome-wide association studies (GWAS) in diverse natural populations have been widely used to characterize genetic architecture but are limited by a fundamental tradeoff when causative variants (*i.e.*, the oligogenic component) are confounded with polygenic variation. Models without population and/or kinship terms partition the colinear variance into the monogenic/oligogenic term (leading to false positive associations) while models with population and/or kinship terms partition colinear variation into polygenic terms (leading to false negatives) (Bergelson and Roux 2010). In a nested association mapping (NAM) population, controlled crosses between the common parent and diverse founders breaks up population structure, increasing power for QTL detection (Myles *et al.* 2009). In addition, the larger population size in most NAM populations mitigates the Beavis effect, the overestimation of QTL effect size that occurs in small populations (Utz *et al.* 2000). NAM has greatly facilitated the characterization of genetic architecture in species where controlled crosses are feasible, including many major crops (Buckler *et al.* 2009; Maurer *et al.* 2015; Bajgain *et al.* 2016; Bouchet *et al.* 2017).

Inflorescence morphology is a key component of crop adaptation and yield (Harlan and de Wet 1972; Cooper *et al.* 2014). Homologous variation of inflorescences among cereals has long been noted (Vavilov 1922) and inflorescence morphology has been a valuable system to investigate the evolutionary dynamics and molecular basis of genetic architecture in plants (Hermann and Kuhlemeier 2011; Zhang and Yuan 2014). Analysis of inflorescence mutants has revealed regulatory networks with genes controlling hormonal biosynthesis, hormone transport, signal transduction, and transcriptional regulation (Zhang and Yuan 2014). Comparative studies indicate that components of inflorescence regulatory networks are largely conserved across grass species, but that substantial variation in ancestral regulatory networks exists within and among species (Kellogg 2007; Barazesh and McSteen 2008; Tanaka *et al.* 2013; Huang *et al.* 2017). However, since most inflorescence regulators were identified via mutant screens, the role of these ancestral genes in natural variation or adaptive divergence of inflorescence morphology is not well understood (Brown *et al.* 2011; Crowell *et al.* 2016; Wu *et al.* 2016). In addition, studies of natural variation may reveal genes not yet identified via mutant analysis.

Sorghum is a source of food, feed, and bioenergy in many parts of the world, especially important to smallholder farmers in semi-arid regions (National Research Council 1996). Sorghum has diffused to contrasting agroclimatic zones, and harbors abundant variation in traits such as height, leaf architecture, and inflorescence morphology. Variation in inflorescence morphology is thought to underlie yield components (Brown *et al.* 2006; Witt Hmon *et al.* 2013) and local adaptation to agroclimatic zones defined by precipitation gradients (Harlan and de Wet 1972; Kimber *et al.* 2013). The five major botanical races of sorghum are largely defined based on inflorescence morphology, along with seed and glume shape (Harlan and de Wet 1972). For instance, guinea sorghums with long open panicles predominate in humid zones while durra sorghums with short compact panicles predominate in arid zones (Kimber *et al.* 2013). A few studies have mapped inflorescence traits in sorghum, but biparental mapping was limited by low diversity and GWAS were limited by confounded population structure (Brown *et al.* 2006; Morris *et al.* 2013; Witt Hmon *et al.* 2013; Olatoye *et al.* 2018). However, the genetic architecture of inflorescence morphology remains poorly understood and none of the underlying natural variants have been cloned in this species. In this study we took advantage of a global NAM resource to provide a more comprehensive view of the genetic architecture of inflorescence morphology in sorghum. Our findings suggest that global sorghum inflorescence variation is under the control of oligogenic, epistatic, and pleiotropic loci, consistent with the Fisher-Orr model under disruptive selection.

## Materials And Methods

### Plant materials and phenotyping

The sorghum NAM population was derived from a cross between an elite U.S. common parent RTx430 and 10 diverse founders that originated from different agroclimatic zones, thereby capturing a wide genetic and morphological diversity (Supplementary Table 1, Supplementary Figure 1) (Bouchet *et al.* 2017). Each diverse parent and its RILs represent a family of 200–233 RILs making a total of 2220 RILs in the population. To represent a typical range of growing conditions, field phenotyping experiments were conducted under rainfed conditions in semi-arid (Hays, Kansas; Agricultural Research Center; 38.86, -99.33) and humid-continental (Manhattan, Kansas; Agronomy North Farm; 39.21, -96.59) environments for two years (2014 and 2015). In Hays in 2015, the NAM RILs were evaluated at two contrasting sites; an upland site that tends to be water-limited (HD15) and a bottomland site that tends to be well-watered (HI15). Each site-by-year was regarded as one environment (Table 1). In the second year (2015), RILs were randomized within maturity blocks of families in a row-column design based the first-year flowering data. Each row (corresponding to a plot) was 3 m with 1 m alleys between ranges.

**Table 1:** Summary of field experiments using the nested association mapping population

Location	Climate*^a^*	Year	Precipitation (mm)*^b^*	Code
Manhattan, KS	Humid Continental	2014	698	MN14
Hays, KS (Upland)	Semi-Arid	2014	639	HA14
Manhattan, KS	Humid Continental	2015	998	MN15
Hays, KS (Bottomland)	Semi-Arid	2015	513	HI15
Hays, KS (Upland)	Semi-Arid	2015	513	HD15

a Koppen-Geiger climate classification for the location.

b Annual precipitation, for October of the prior year to October of the given year. (National Oceanic and Atmospheric Administration, U.S. Department of Commerce.)

The NAM RILs were phenotyped at F6:7 and F6:8 generations for upper primary branch length (UBL), lower primary branch length (LBL), rachis length (RL), and rachis diameter (RD) (Supplementary Figure 2). Three random panicles were collected from each plot after physiological maturity and subsequently used for phenotyping. Inflorescence morphology traits were measured using barcode rulers (1 mm precision) and barcode readers (Motorola Symbol CS3000 Series Scanner, Chicago IL, USA). RL was measured as the distance from the apex of the panicle to the point of attachment of the lowest rachis lower primary branch (Brown *et al.* 2006). RD was measured using a digital Vernier caliper (0.1 mm precision) as the diameter of the peduncle at the point of attachment of the bottommost rachis lower primary branch. For UBL, three primary branches were randomly detached from the apex of the panicle. For LBL, three primary branches were randomly detached from the region closest to the peduncle for two panicles (Supplementary Figure 2).

### Genomic data analysis

Genotyping-by-sequencing of the NAM population and diverse global germplasm was previously described (Bouchet *et al.* 2017; Hu *et al.* 2019). Briefly, Illumina sequence reads were aligned to the BTx623 reference genome version 3 using Burrow Wheeler Aligner 4.0 and SNP calling was done using TASSEL-GBS 5.0 (Glaubitz *et al.* 2014). For the current study, missing data imputation was done in two stages using Beagle 4 (Browning and Browning 2013). The NAM population and the sorghum association mapping population (SAP) GBS data were first extracted from the build. Filtering was conducted to remove markers with (i) tri-allelic SNPs, (ii) missing data in more than 80% of individuals, or (iii) < 3% minor allele frequency prior to imputation. The NAM population and sorghum association panel (SAP; 334 accessions) (Casa *et al.* 2008) were imputed separately and each germplasm set was filtered for MAF > 0.05. NAM RILs with >10% heterozygosity were dropped from the analysis.

### Phenotype and heritability analysis

Phenotypic data analysis was carried out using R programming language and SAS (SAS Institute Inc., Cary, NC, USA). All traits were tested for normality and the only trait (UBL) with significantly skewed distribution was log transformed. Analysis of variance was performed for each trait using *aov* function in R. The best linear unbiased prediction (BLUP) of each trait was estimated using data from five environments with *lmer* function in *LME4* package in R (Bates *et al.* 2015) with genotype, environment, and genotype-environment interactions fitted as random effects (Wu *et al.* 2016). The variance components used for broad sense heritability (*H*^2^) were estimated using the maximum likelihood method by PROC VARCOMP of the SAS software (SAS Institute Inc., Cary, NC, USA). RIL-nested-within-family and RIL-nested-within-family by environment interaction were fit as random effects. The resulting variance components were used to estimate the broad sense heritability (*H*^2^) following equation 1 in (Hung *et al.* 2012) as:

$$H^{2}=\frac{{\hat{\sigma }}_{RIL{\left(family\right)}_{p}}^{2}}{{\hat{\sigma }}_{RIL{\left(family\right)}_{p}}^{2}+\frac{{\hat{\sigma }}_{env*RIL{\left(family\right)}_{p}}^{2}}{n_{{envl}_{p}}}}+\frac{{\hat{\sigma }}_{\varepsilon }^{2}}{n_{{plot}_{p}}}$$ [1]

where ${\hat{\sigma }}_{RIL{\left(family\right)}_{p}}^{2}$ is the variance component of RILs nested within family *p*, $n_{envl_{p}}$ is the harmonic mean of the number of environments in which each RIL was observed, and $n_{plot_{p}}$ the harmonic mean of the total number of plots in which each RIL was observed. Pearson pairwise correlation between traits was estimated using the residuals derived from fitting a linear model for family and trait phenotypic means:

$$y=\mu +\gamma_{\text{i}}+\varepsilon_{ij}$$ [2]

where **y** is the vector of phenotypic data, μ is the overall mean, γ_i_ is the term for the NAM families, and ε*_ij_* is the residual.

### Joint linkage mapping

Joint linkage analysis was performed using 92,391 markers and 2220 RILs. This approach is based on forward inclusion and backward elimination stepwise regression approaches implemented in TASSEL 5.0 stepwise plugin (Glaubitz *et al.* 2014). The family effect was accounted for as a co-factor in the analysis. First, a nested joint linkage (NJL) model was fitted where markers were nested within families (Poland *et al.* 2011; Würschum *et al.* 2012). In addition, a non-nested joint linkage model (JL), where markers were not nested within families, was used due to its higher predictive power than NJL (Würschum *et al.* 2012). Entry and exit F_test_ values were set to 0.001 and based on 1000 permutations, the *P*-value threshold was set to 1.84 × 10^−6^. The JL model was specified as:

$$y=b_{o}+\alpha_{f}u_{f}+\sum_{i=1}^{k}x_{i}b_{i}+e_{i}$$ [3]

where *b_0_* is the intercept, *u_f_* is the effect of the family of founder *f* obtained in the cross with the common parent (RTx430), α*_f_* is the coefficient matrix relating *u_f_* to *y*, *b_i_* is the effect of the *i*^th^ identified locus in the model, *x_i_* is the incidence vector that relates $b_{i}$ to *y* and *k* is the number of significant QTL in the final model (Yu *et al.* 2008).

### Genome-wide association studies

GWAS was performed for all traits using 92,391 markers and 2220 RILs using BLUPs adjusted by environments. The multi-locus-mixed model (MLMM) approach (Segura *et al.* 2012) implemented in R was used for GWAS in the NAM population, as described previously (Bouchet *et al.* 2017). The MLMM approach performs stepwise regression involving both forward and backward regressions, accounts for major loci and reduces the effect of allelic heterogeneity. The family effect was fitted as a co-factor and a random polygenic term (kinship relationship matrix) was also accounted for in the MLMM model. Bonferroni correction with α = 0.05 was used to determine the cut-off threshold for each trait association (α**/**total number of markers = 5.4 × 10^−7^).

For comparison with NAM, GWAS was performed in the SAP using general linear model (GLM) and compressed mixed linear model (CMLM) with the GAPIT R package (Lipka *et al.* 2012) to match a previous study (Morris *et al.* 2013). The GLM (naive model) did not account for population structure and was specified as:

y=Sα+e [4]

where **y** is the vector of phenotypes, α is a vector of SNPs effects, and *e* is the vector of residual effects, and **S** is the incident matrix of 1s and 0s relating *y* to α. The CMLM model (full model) accounted for population structure and polygenic background effects (kinship) was specified as:

y=Sα+Qv+Zu+e [5]

where **y** is the vector of phenotype, and *u* is a vector of random genetic background effects. **X**, **Q**, and **Z** are incident matrices of 1s and 0s relating **y** to β and *u* (Yu *et al.* 2006). The phenotypic data in the SAP used for GWAS is from a previous study (Brown *et al.* 2008; Morris *et al.* 2013). A custom script was used to identify QTL that overlapped within a 50 kb window (a conservative LD window with regards to LD decay in sorghum NAM and SAP) (Hu *et al.* 2019) between the NAM and GWAS (GLM or CMLM) mapping results for LBL and RL.

### Effect size and allele frequency estimation

Allele frequencies at the SNPs were calculated using snpStats package in R (Clayton 2014). The additive effect size of QTL within and across families were estimated as the difference between the mean of the two homozygous classes for each QTL divided by two. The additive effect of each QTL was estimated relative to RTx430. The sum of squares of QTL divided by the total sum of squares gave the proportion of variance explained. To estimate within-family variation explained by each QTL, a regression model was fit with terms for family and QTL nested within family as fixed effects (Würschum *et al.* 2012):

$$y_{ijkl}=\mu +\gamma_{i}+\omega_{jk}+\varepsilon_{ijkl}$$ [6]

where *y_ijkl_* is the phenotype, γ_i_ is the family term, ω*_jk_* is the term for QTL nested within family, and ε*_ijk_* is the residual.

### Grass homologs search around identified loci and enrichment analysis

A set of known genes that control inflorescence morphology in grasses was compiled from literature consisting of 20 maize genes, eight rice genes, and one foxtail millet gene; in addition two sorghum genes that control plant architecture were included (number of genes = 30; Supplementary File 1). Based on this candidate gene set, 138 sorghum homologs (orthologs and paralogs) were downloaded from Phytozome 12, which uses mutual best hit and Hidden Markov Model peptide profiles to identify putative homologs (Goodstein *et al.* 2012). To identify putative orthologs, the most similar homolog was identified, then orthology was confirmed based on synteny using the “Gene Ancestry” synteny viewer in Phytozome and (for maize genes) the Classical Maize Genes browser (Schnable and Freeling 2011). For four genes where the most similar homolog does not have synteny evidence, the homology type is denoted as “Most similar homolog” in Supplementary File 1. All putative orthologs mentioned in the text are most similar and syntenic with the known maize, rice, or foxtail millet gene. A custom R script was used to search for homologs within 150 kb window upstream and downstream of each association, based on the LD decay rate in the NAM population (Hu *et al.* 2019). Enrichment analysis of *a priori* genes around identified QTL was performed using chi square test to compare observed colocalization frequency with colocalization of QTL with random genes from the sorghum genome version 3.1 gff3 file on Phytozome. Descriptions of expression patterns for candidate genes are based on the Phytozome gene expression atlas, which covers 47 RNA sequencing profiles for various tissues and treatments.

### Geographic analysis of SNPs at inflorescence QTL

For three inflorescence morphology QTL that colocalized with sorghum orthologs of maize or rice inflorescence genes, the geographic distribution of the SNP alleles was investigated. Allelic data for the targeted SNPs was extracted from GBS SNP data for global georeferenced sorghum accession (number of accession = 2,227; number of SNP = 431,691) (Hu *et al.* 2019). The alleles were then plotted on a global geographic map with national boundaries based on the geographic coordinates of each georeferenced accession. Climatic association test was performed for targeted SNPs between the annual mean precipitation and allelic variation in georeferenced global accessions using both the naive model (GLM) and the mixed model that accounted for kinship only.

### Data availability

Phenotype and genotype data are available at FigShare: https://figshare.com/s/ae874edd86775a9d1b1d. File S1 contains detailed descriptions of QTL information, *a priori* gene list and *a priori* genes that colocalized with QTL. File S2 contains heatmap of QTL effects within NAM families. File S3 contains detailed description of associations that colocalized between NAM, GLM, and CMLM and results of association of inflorescence QTL alleles with precipitation for both GLM and MLM. The NAM population seeds are available from the USDA National Plant Germplasm System (https://www.ars-grin.gov/). Raw sequencing data for the NAM population are published (Bouchet *et al.* 2017) and available in the NCBI Sequence Read Archive under project accession SRP095629 and on Dryad Digital Repository (https://doi:10.5061/dryad.gm073). R scripts and Linux shell scripts are available at https://github.com/marcbios/Sorghum-Inflorescence-Nested-Association-Mapping. Supplemental material available at figshare: https://doi.org/10.25387/g3.11356274.

## Results

### Variation of inflorescence morphology in the NAM population

**Figure 1:**
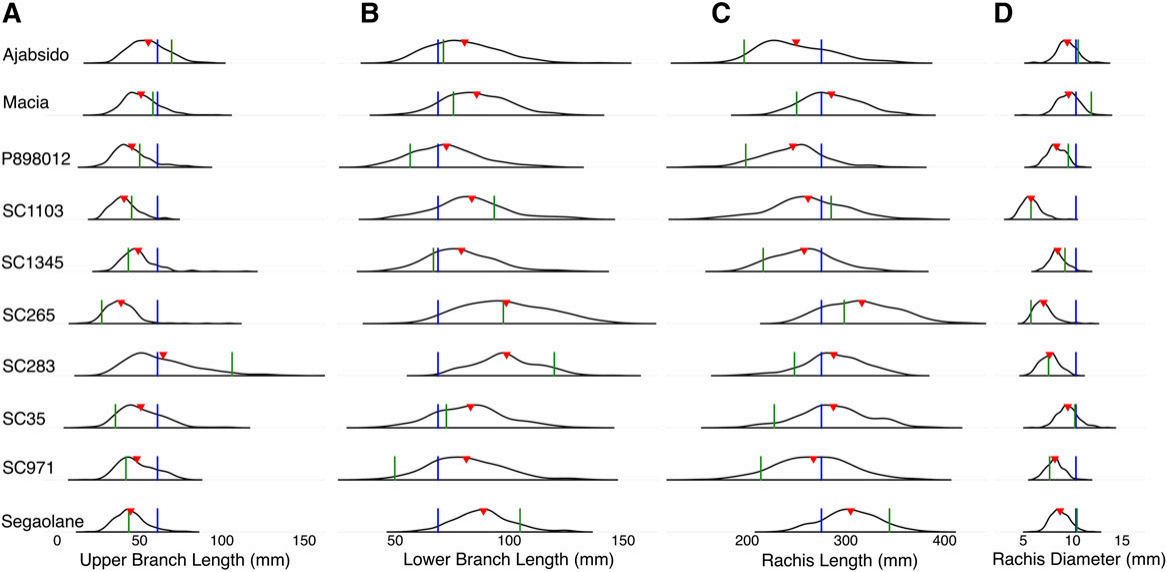
Phenotypic distribution of sorghum inflorescence morphology. Phenotypic distribution of line means for each recombinant inbred line (RIL) family and each of the inflorescence traits, (A) lower branch length, (B) upper branch length, (C) rachis length, and (D) rachis diameter. Blue lines indicate mean trait value for the common parent (RTx430), green lines indicate mean trait values for each of the other parents (listed on the left), and red triangles indicate the mean trait value across the RILs for each family.

**Figure 2:**
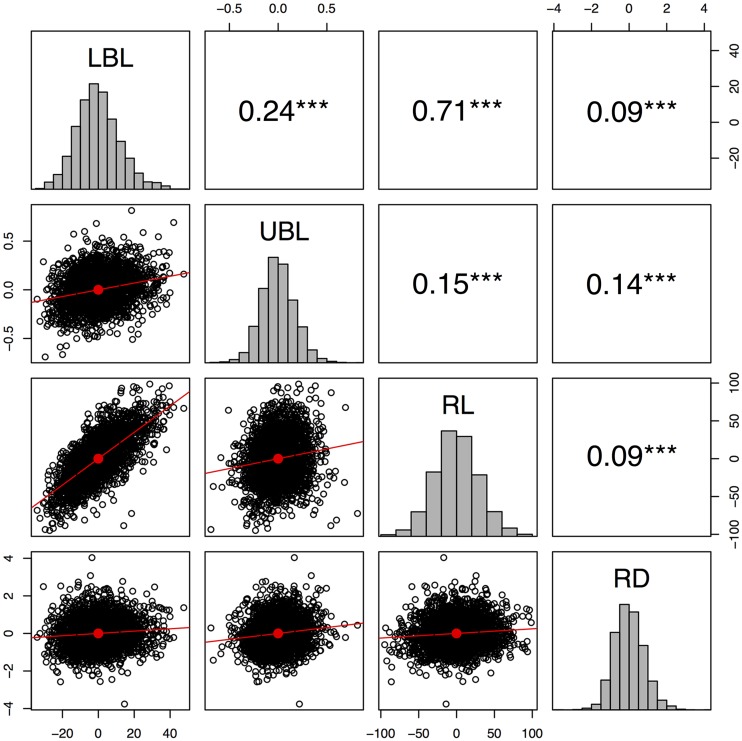
Pairwise correlation among inflorescence morphology traits. Pearson correlation (upper diagonal) between residuals of the regression of the family on the best linear unbiased predictors (BLUPs) of lower branch length (LBL), upper branch length (UBL), rachis length (RL), and rachis diameter (RD). Significance at 0.001 (***) is noted. BLUPs were estimated across five environments (site-by-year).

Phenotypic measurements were collected for four inflorescence morphology traits across five environments (Table 1; Figure S2), representing over 198,000 observations. The number of RILs in each family ranged from 202 in the Segaolane family to 233 in the SC265 family (Supplementary Table 1). Significant genotypic differences were observed for all four inflorescence traits (Table 2). The broad-sense heritability estimates for all four traits were high, ranging from 0.59 to 0.92. The SC265 and SC283 families had the longest lower branches (mean across RILs of 99 mm). The SC283 family had the longest upper branches (mean across RILs of 64 mm). The SC265 and Segaolane families had the longest rachis, with mean lengths of 316 mm and 305 mm, respectively. The largest rachis diameters were observed in the Ajabsido, Macia, and SC35 families (a mean of ∼9.5 mm across RILs in each family). Phenotypic variation distribution within families showed that in some families the mean trait value of the RILs was greater than the mean of either parent (Figure 1). The highest trait-by-trait phenotypic correlations were for RL and LBL (*r* = 0.71; *P*-value < 0.01). By contrast, UBL and LBL had a low positive correlation (*r* = 0.19; *P*-value < 0.01), and RL had no correlation with either UBL or RD (Figure 2).

**Table 2:** Mean, range, and broad sense heritability (*H*^2^) for lower branch length (LBL), upper branch length (UBL), rachis length (RL), and rachis diameter (RD)

Trait*^a^*	Range (mm)	Mean (mm)	*H*^2^
LBL***	267 – 176	82	0.86
UBL*	7 – 170	48	0.85
RL***	111 – 465	274	0.92
RD***	3.8 – 13.5	8.3	0.59

a Significant genotypic differences given by *, **, *** at 0.05, 0.01 and 0.001, respectively.

### QTL variation in the NAM population

**Figure 3:**
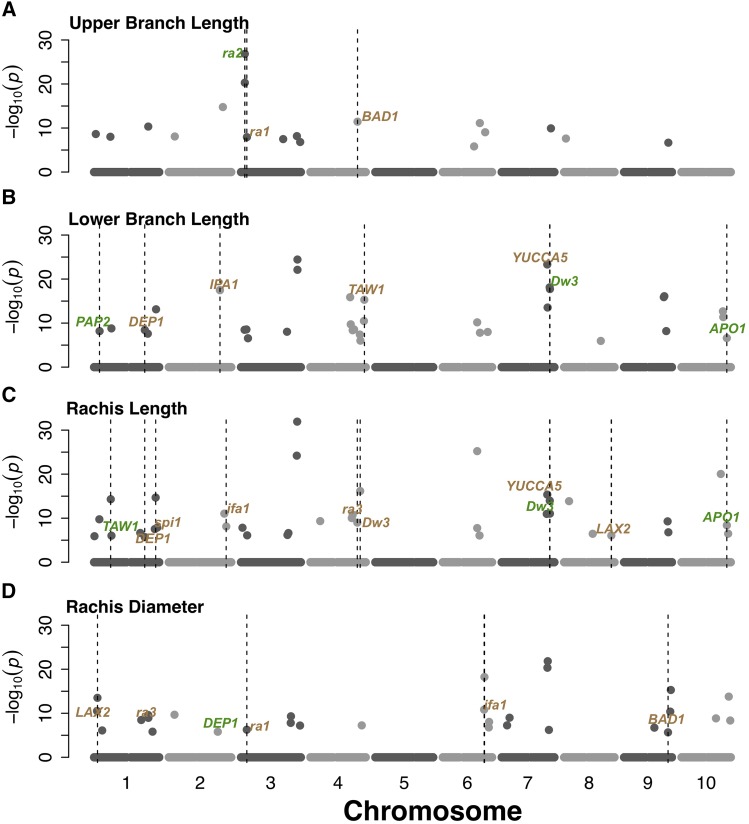
QTL mapping for inflorescence morphology using joint linkage model. Genome positions of loci associated with (A) upper branch length, (B) lower branch length, (C) rachis length, and (D) rachis diameter. *A priori* candidate genes that colocalize with QTL within 150 kb are noted as follows. Green text indicates putative sorghum orthologs of *a priori* candidate genes while brown text indicates paralogs.

A total of 116,405 SNPs were obtained after SNP calling, imputation, and filtering (minimum MAF = 5%). After filtering for 0.96 inbreeding coefficient, a total of 92,391 markers were identified. Significant QTL associations were observed for all traits when using MLMM, JL, and NJL models (Figure 3, Supplementary Figure 3–4). MLMM identified nine significant associations in total for all traits. The JL model identified 81 QTL, while the NJL model identified 40 QTL across all traits (Supplementary File 1 and Supplementary Table 2). Allele frequencies at the QTL ranged from 0.05 to 0.48 (Supplementary File 1). The proportion of within-family variation explained by all QTL (*i.e.*, an estimate of the oligogenic component) varied substantially among traits, with 12%, 37%, 31%, and 21% of variation explained by QTL for UBL, LBL, RL, and RD, respectively. Within-family and across-family effects of each QTL for NJL and JL models were estimated relative to RTx430 (Supplementary File 2). LBL QTL qSbLBL7.5960 explained the largest proportion of variation (4.6%) among all QTL identified in this study (Table 3).

**Table 3:** Inflorescence QTL that explain > 1.5% of variation across the NAM population

QTL code	MAF*^a^*	PVE*^b^*	Trait*^c^*	Gene colocalization*^d^*	QTL colocalization*^e^*
qSbUBL3.0475	0.18	3.8	UBL	Yes (*ra2* ortholog)	Brown *et al.* 2006
qSbUBL3.0476	0.26	3.2	UBL	Yes (*ra2* ortholog)	Brown *et al.* 2006
qSbUBL2.6719	0.43	2.1	UBL		
qSbUBL3.0734	0.23	1.8	UBL	Yes (*ra1* paralog)	
qSbUBL6.4606	0.11	1.7	UBL		
qSbUBL3.5243	0.05	1.6	UBL		
qSbRL10.4877	0.39	2.6	RL		
qSbRL3.6985	0.32	2.5	RL		
qSbRL6.4280	0.23	2.2	RL		
qSbRL4.5134	0.16	1.8	RL		
qSbRL7.5975	0.18	3.0	RL	Yes (*Dw3/YUCCA5*)	Brown *et al.* 2006
qSbRL1.7845	0.36	1.7	RL		
qSbRL1.2156	0.38	1.5	RL		
qSbRL3.6936	0.44	1.5	RL		
qSbRL6.4277	0.32	1.5	RL		
qSbLBL7.5975	0.18	4.4	LBL	Yes (*Dw3/YUCCA5*)	Brown *et al.* 2006
qSbLBL7.5960	0.18	4.6	LBL		
qSbLBL7.5663	0.23	4.3	LBL		
qSbLBL7.5692	0.18	3.6	LBL		
qSbLBL4.5244	0.1	3.1	LBL		
qSbLBL4.4933	0.41	2.8	LBL		
qSbLBL4.6693	0.05	2.6	LBL		
qSbLBL4.6210	0.17	2.5	LBL		
qSbLBL4.5005	0.47	2.3	LBL		
qSbLBL2.6358	0.26	2.2	LBL		
qSbLBL7.5995	0.43	1.8	LBL	Yes (*Dw3*)	Brown *et al.* 2006
qSbLBL10.5188	0.13	1.8	LBL		
qSbLBL2.6358	0.43	1.7	LBL	Yes (*IPA1* paralog*)*	
qSbLBL4.5421	0.3	1.7	LBL		
qSbLBL3.7019	0.37	1.6	LBL		
qSbLBL7.5707	0.34	1.6	LBL		
qSbLBL9.4934	0.38	1.6	LBL		
qSbLBL3.7019	0.37	1.5	LBL		

a MAF: Minor allele frequency.

b PVE: Proportion of variation explained.

c Lower branch length (LBL), upper branch length (UBL), rachis length (RL), and rachis diameter (RD).

d Denotes if there is a colocalization with *a priori* candidate gene (within 150 kb from QTL). Details on colocalized genes are provided in Table 4.

e Denotes if there is a colocalization with a QTL from a previous biparental linkage study (Brown *et al.* 2006) or GWAS (Morris *et al.* 2013).

### QTL colocalization and enrichment with *a* priori candidate genes

To assess the overall role of variation at ancestral inflorescence regulators, we performed colocalization and enrichment analysis between the QTL and a set of *a priori* candidate genes containing sorghum homologs of rice, maize, and foxtail millet genes (n = 138).

NAM QTL were significantly enriched for colocalization with *a priori* candidate genes (2-fold enrichment; *P*-value < 0.001). Of 123 unique QTL, 28 colocalized with *a priori* genes. Among the QTL that overlapped with *a priori* candidate genes, two QTL were inside the gene, three QTL were <15 kb from the gene, 16 unique QTL were 15–100 kb from the gene, and eight unique QTL were 100–150 kb from the genes (Table 4 and Supplementary File 1). Overall, 24 genes colocalized with inflorescence QTL, while 114 *a priori* candidate genes (of 138) did not overlap with any inflorescence QTL (Supplementary File 1).

**Table 4:** Details on QTL that colocalize with *a priori* candidate genes

QTL ID*^a^*	MAF*^b^*	PVE*^c^*	Trait*^d^*	Gene Name	Sorghum ID*^e^*	% Sim*^f^*	Homology	Proximity (kb)*^g^*
qSbLBL7.5975	0.18	4.4	LBL	*YUCCA5**^h^*	Sobic.007G163200	62	Paralog	In gene
qSbRL7.5975	0.18	3.0	RL	*YUCCA5**^h^*	Sobic.007G163200	62	Paralog	In gene
qSbUBL3.0475	0.18	3.8	UBL	*Ramosa2 (ra2)**^h^*	Sobic.003G052900	92.7	Ortholog	38
qSbUBL3.0476	0.26	3.2	UBL	*Ramosa2 (ra2)**^h^*	Sobic.003G052900	92.7	Ortholog	32
qSbLBL2.6358	0.26	2.2	LBL	*Ideal Plant Architecture (IPA1)*	Sobic.002G247800	64.3	Paralog	In gene
qSbUBL3.7343	0.23	1.8	UBL	*Ramosa1 (ra1)*	Sobic.003G084400	14.3	Paralog	87
qSbLBL7.5995	0.43	1.8	LBL	*Dwarf3 (Dw3)**^h^*	Sobic.007G163800		Known gene	131
qSbLBL2.6348	0.43	1.7	LBL	*Ideal Plant Architecture (IPA1)*	Sobic.002G247800	64.3	Paralog	101
qSbRL1.2067	0.45	1.4	RL	*TAWAWA1 (TAW1)*	Sobic.001G219400	70.1	Ortholog	112
qSbRL1.7649	0.42	1.4	RL	*sparse inflorescence1 (spi1)*	Sobic.001G495850	70.4	Paralog	70
qSbUBL4.5850	0.31	1.4	UBL	*BRANCH ANGLE DEFECTIVE 1 (BAD1)*	Sobic.004G237300	11.6	Paralog	15
qSbRL10.5631	0.42	1.2	RL	*Aberrant Panicle Organization (APO1)**^i^*	Sobic.010G220400	89.9	Ortholog	58
qSbRL1.6301	0.1	1.1	RL	*DENSE AND ERECT PANICLE (OsDEP1)*	Sobic.001G341700	14.8	Paralog	101
qSbRD6.5177	0.22	1.0	RD	*indeterminate floral apex1 (ifa1)*	Sobic.006G160800	38.2	Paralog	95

a Quantitative trait loci identification (QTL ID)

b MAF: minor allele frequency.

c Proportion of variation explained (PVE) >=1.0%

d Lower branch length (LBL), upper branch length (UBL), rachis length (RL), and rachis diameter (RD).

e Sorghum homolog.

f Percentage similarity of sorghum gene to reference gene.

g Proximity of SNP from joint linkage mapping QTL to nearest *a priori* candidate gene.

h Gene identified previously by Brown *et al.* 2006.

i Gene identified previously by Morris *et al.* 2013.

### Comparison of NAM and diversity panel GWAS

**Figure 4:**
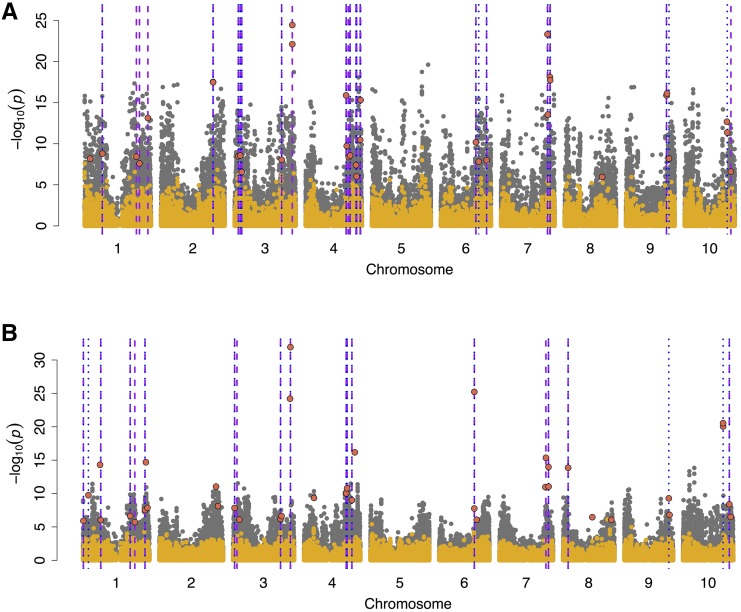
Comparison of joint linkage in a NAM population *vs.* genome-wide association in a diversity panel. Manhattan plot for the comparison of genome-wide association approaches for (A) lower branch length and (B) rachis length using general linear model (GLM) in gray, compressed mixed linear model (CMLM) in yellow, and NAM joint linkage (JL) model in red. Broken lines in purple and blue note colocalization between NAM and GLM (50 kb window), and between NAM and CMLM (50 kb window), respectively. GLM and CMLM were carried out in the sorghum association panel (SAP, n = 334) and NAM (n = 2200).

NAM provides an independent approach to validate GWAS QTL from diversity panels and assess the relative performance of GWAS models. We compared the inflorescence loci identified in the NAM with GWAS QTL for LBL and RL identified in the SAP, identifying colocalization (within 50 kb) between NAM QTL SNPs and top 5% SNP associations in the GLM or CMLM (Figure 4 and Supplemental File 2). For LBL, the comparison revealed 26 overlaps between NAM *vs.* GLM, and 20 overlaps between NAM *vs.* CMLM. For RL, the comparison revealed 17 overlaps for both NAM *vs.* GLM and NAM *vs.* CMLM. To identify gene candidates that are supported by multiple mapping approaches, *a priori* candidate genes were cataloged in overlapping NAM and GWAS QTL (Supplementary File 3). For LBL, five *a priori* candidate genes colocalized with overlapping NAM and GLM QTL, while two *a priori* candidate genes colocalized with overlapping NAM and CMLM QTL. Similarly for RL, six *a priori* candidate genes colocalized with overlapping NAM and GLM QTL, and six genes colocalized with overlapping NAM and CMLM QTL.

### Geographic distribution of allele and environment-marker associations

**Figure 5:**
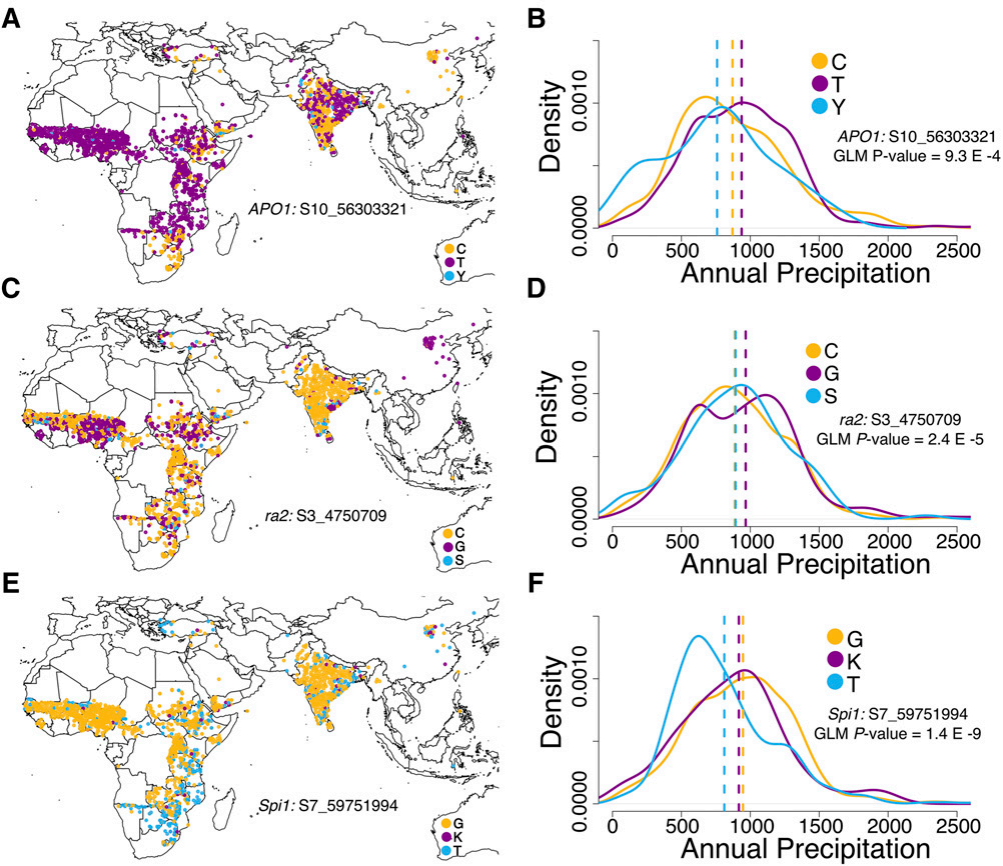
Global geographic allele distribution at some inflorescence QTL discovered in the NAM population. Global geographic and precipitation gradient distribution of alleles atSNP S10_56303321 associated with lower branch length that colocalized the sorghum ortholog of rice *Aberrant Panicle Organization1* (A-B), SNP S3_4750709 associated with lower branch length that colocalized the sorghum ortholog of maize *ramosa2* (C-D), and SNP S7_59751994 associated with upper branch length that colocalized the sorghum ortholog of maize *sparse inflorescence1* (E-F). Dashed lines in density plots represent the mean of each distribution.

For three NAM QTL that were near *a priori* candidate genes, we investigated the SNP allele distribution in global georeferenced accessions (Figure 5). Strong geographic patterns were observed for SNP alleles associated with inflorescence morphology variation, though the patterns differed among SNPs (Figure 5 A, C, and E). The LBL-associated C allele (S10_56303321) near the sorghum ortholog of *APO1* was predominant in Horn of Africa, Yemen, southern Africa, southern India, and China. The T allele was predominant in west and central Africa. For the UBL-associated SNP (S3_4750709) near *ramosa2*, the C allele was predominant in most of Africa and India, while the G allele was predominant in Nigeria, Sierra Leone, and China. For the LBL-associated SNP (S7_59751994) near *YUCCA5* (*i.e.*, the *sparse inflorescence1* paralog), one allele was predominant in west Africa and India, while the other allele was predominant in southeastern Africa. Based on GLM the differentiation of the alleles across precipitation gradient (considering annual precipitation as a proxy) was nominally significant, but weak, for all three SNPs (Figure 5 B, D, and F). None were significantly differentiated under a MLM that accounted for kinship (File S3).

## Discussion

### Genetic architecture of inflorescence adaptation

Nested association mapping can help characterize the genetic architecture of adaptive traits while avoiding some pitfalls of GWAS. This sorghum NAM study provides a high-powered dissection of genetic architecture for global variation in inflorescence morphology, a key trait for adaptation across agroclimatic zones (Harlan and de Wet 1972; Olatoye *et al.* 2018). Our study identified many new loci (Table 3) and provided more precise mapping of known loci (Brown *et al.* 2006). Among the known QTL is LBL QTL (qSbLBL7.5975), which appears to be pleiotropic with RL (as qSbRL7.5975). Previous linkage mapping studies identified association around this same *Dw3* region for QTL associated with rachis length and primary branch length (Brown *et al.* 2006; Shehzad and Okuno 2015) and *YUCCA5* was proposed as a candidate gene for the branch length QTL (Brown *et al.* 2008).

The preponderance of moderate and large effect QTL for four inflorescence morphology traits suggests a predominantly oligogenic trait architecture for inflorescence variation in global sorghum diversity (Supplementary Table 2, Supplementary Figure 3-4, Supplementary File 1-2). Note, a PVE estimate that would be considered “small effect” (*e.g.*, 1%) in a typical biparental study (*e.g.*, 100–300 RILs) may be better characterized as “moderate effect” in a NAM population, given the denominator is phenotypic variance across many diverse families and thousands of RILs. In previous studies of sorghum inflorescence in biparental populations, large effect loci (explaining up to 19% of the variance) were found, but effect size of these loci may have been upwardly biased due to the Beavis effect (Xu 2003). The population size of the NAM (2200 RILs) used in this study should provide a more robust estimation of QTL effect size, which are expected to be accurate with population sizes >1000 (King and Long 2017). In maize, effect size distribution of loci associated with ear and tassel traits has been linked to strong directional selection during maize domestication (Brown *et al.* 2011; Xue *et al.* 2016). In sorghum, the moderate to large effect loci identified here may reflect selection toward multiple contrasting fitness optima during the adaptation to contrasting agroclimatic zones, consistent with the Fisher-Orr model under disruptive selection (Orr 2005; Tenaillon 2014).

Epistasis may be reflected in asymmetric transgressive variation (Rieseberg *et al.* 1999; Gaertner *et al.* 2012). The shift of the RIL means from the mid-parent value in some families, and some strongly skewed trait distributions in NAM RILs, suggest that epistasis may be pervasive (*e.g.*, UBL in SC283 family or RD in SC1103 family; Figure 1). These results support previous findings of pervasive epistasis for inflorescence and plant morphology traits in a diallel population in sorghum (Ben-Israel *et al.* 2012). Further evidence for epistatic interactions of additive QTL can be provided by opposite allelic effects of QTL across families (Buckler *et al.* 2009; Peiffer *et al.* 2014). Consistent with a hypothesis of gene-by-genetic background epistasis, inflorescence morphology QTL showed opposed allelic effects across families for 63% (82/131) of QTL (Supplementary File 2). Other QTL (16%) identified had consistent allelic effects in all families. These loci may influence inflorescence variation additively across multiple botanical races, or may reflect rare variants in the common parent. Further analyses to map interacting loci will be needed to characterize the role of epistasis in sorghum inflorescence variation (Chen *et al.* 2019).

Genetic correlation among traits due to linkage or pleiotropy can limit or promote adaptive evolution (Lynch and Walsh 1998). LBL and RL had high phenotypic correlation (*r* = 0.71, *P*-value < 0.001) and had two major effect QTL that were in common (qSbLBL7.5975/qSbRL7.5975 and qSbLBL10.5630/qSbRL10.5631) (Figure 3). Given the large size of the NAM population, and concomitant high mapping resolution, if these QTL colocalizations are not due to pleiotropy then linkage must be very tight (*e.g.*, <2 cM). In maize, mutations in the *YUCCA*-family gene *sparse inflorescence1* led to drastic reduction in both inflorescence rachis length and branch length (Gallavotti *et al.* 2008), suggesting pleiotropy as a parsimonious explanation for the genetic correlation of LBL and RL. By contrast, the two branch length traits (LBL and UBL) had relatively low phenotypic correlation (0.24) and lack of colocalization between QTL, suggesting that they are largely under independent genetic control. Studies of the underlying molecular network (*e.g.*, mutant analysis, spatiotemporal expression dynamics) should provide further insight on the basis of pleiotropic *vs.* independent genetic control (Eveland *et al.* 2014).

Studies have shown evidence of local adaptation across agroclimatic zones for several sorghum traits (Lasky *et al.* 2015; Olatoye *et al.* 2018; Wang *et al.* 2020). For sorghum inflorescence, there is evidence from phenotypic correlations of clinal adaptation across a regional precipitation gradient (Olatoye *et al.* 2018). In this study, we observed differing global geographic distribution of the alleles at inflorescence QTL that colocalized with *a priori* genes regulating inflorescence branch traits like lower branch length and upper branch length (Figure 5). This finding is similar to previous reports (based on GWAS and geographic allele distribution in a smaller georeferenced panel) suggesting the spread of multiple alleles influencing inflorescence traits (Morris *et al.* 2013). However, the inflorescence QTL alleles were not strongly associated with annual mean precipitation across global precipitation zones (Figure 5). This suggests that the variation at these selected genes may not underlie clinal adaptation of inflorescence to the global precipitation gradient.

Our comparison of NAM and GWAS QTL suggests that naive GWAS models (GLM) can contain valuable associations signals for adaptive traits that may be missed in mixed model association. This inference is based on the finding that the number of *a priori* candidate genes that colocalized with NAM *vs.* GLM overlaps was higher than the number that colocalized with NAM *vs.* CMLM overlaps (Figure 4 and Supplementary File 1). While nominal GLM *P*-values are often inflated, the top associations in simple GLM may reflect true QTL that are not identified in MLM because they are colinear with polygenic variance and accounted for by the polygenic term (Bergelson and Roux 2010; Vilhjálmsson and Nordborg 2013).

### Evidence of variation in ancestral regulatory networks

Conserved regulatory networks underlying inflorescence development have been identified by comparative mutant and QTL studies (Kellogg 2007; Zhang and Yuan 2014). However, it is not yet known whether variation in these ancestral regulatory networks underlies local adaptation of inflorescence morphology. The enrichment of sorghum homologs of grass inflorescence genes at inflorescence QTL suggests that a substantial proportion of sorghum inflorescence variation is due to polymorphism in ancestral regulatory networks that have been elucidated in maize and rice. Still, many of the observed QTL did not colocalize with *a priori* candidate genes (see Table 3), so may be due to genes not previously implicated in inflorescence development. We note that the QTL mapping and gene colocalization studies presented here can generate hypotheses on potential causative genes but not test these hypotheses. Further functional studies, such as fine mapping, mutant analysis, and gene expression analysis, will be required to test hypotheses on potential causative genes (Murphy *et al.* 2014; Jiao *et al.* 2016).

Some of the *a priori* candidate genes that colocalized with inflorescence QTL were sorghum homologs of hormone transporters or biosynthesis enzymes that regulate inflorescence development. One example is at qSbLBL7.5975/qSbRL7.5975, which was centered on the intragenic region of *YUCCA5* (Sobic.007G163200; putative flavin monooxygenase auxin biosynthesis gene) (Figure 3, Supplementary Figure 3-4). This *YUCCA5* gene is a paralog of maize auxin biosynthesis gene *sparse inflorescence1* (*Spi1*; 62% similar to maize *Spi1*) (Figure 3B). However, the sorghum *YUCCA5* gene has little to no expression in the tissues/treatments assayed in the Phytozome expression atlas. The peak SNP for this QTL is also 70 kb from the canonical sorghum height gene and auxin efflux transporter *Dw3* (Sobic.007G163800), which could another candidate to consider (Figure 3B and 3C).

Several other *a priori* candidate genes under QTL are homologs of transcription factors that regulate gene expression during inflorescence meristem differentiation in cereals. For instance, the top UBL QTL (qSbUBL3.0475) colocalized with the sorghum ortholog of maize *ramosa2* (*ra2*) encoding a C2H2 zinc-finger transcription factor (Sobic.003G052900, 92.7% similarity to maize *ra2*). In maize and sorghum, the *ra2* transcript is expressed in a group of cells that predicts the position of axillary meristem formation in inflorescence (Bortiri *et al.* 2006; Eveland *et al.* 2014). The QTL qSbUBL4.5850 colocalized with a putative TCP transcription factor (Sobic.004G237300; 15 kb from the gene) that is a paralog of maize tassel development gene *Branch Angle Defective1* (*BAD1*; 12% similar) (Bai *et al.* 2012). While this distant paralog of *BAD1* is unlikely to have the same function, the expression of Sobic.004G237300 is highest in peduncle and upper internode at floral initiation stage, suggesting it may be an interesting candidate for further study.

An LBL and RL QTL (qSbLBL10.5630/qSbRL10.5631) colocalized with the sorghum ortholog of rice *Aberrant Panicle Organization1* (*APO1*) (Sobic.010G220400, 90% similar to rice *APO1*; 58 kb away) (Figure 3B and 3C). In rice, APO1 encodes an F-box protein that regulates inflorescence meristem fate (Ikeda *et al.* 2007). Sorghum *APO1* was also tagged (inside the gene) by a top branch length-associated SNP in a previous GWAS using the SAP (Morris *et al.* 2013), strongly suggesting this gene underlies variation for inflorescence compactness. Another RL QTL (qSbRL1.2067) colocalized with the sorghum ortholog of rice *TAW1* (*TAWAWA1*) gene (Sobic.001G219400, 70% similar to rice *TAW1*). Based on the Phytozome expression atlas, sorghum *TAW1* transcript is highest in peduncle and internode at floral initiation. Given that TAW1 regulates development of the rice inflorescence meristem (Yoshida *et al.* 2013), the findings suggest the hypothesis that *TAW1* conditions natural variation for inflorescence morphology in grasses more generally.

### Prospects for genome-wide dissection and prediction of inflorescence morphology

This study provided a large-scale characterization of the genome regions that influence inflorescence morphology variation across global sorghum diversity. It is likely that additional variation in inflorescence morphology is yet to be discovered in sorghum, since at least ∼30% of global variation was not captured in the 11 NAM founder parents (Bouchet *et al.* 2017). Therefore, increasing the number of families in the NAM resource should be beneficial for both increased mapping resolution and allelic diversity. Although this may increase phenotyping burden, the use of high-throughput phenotyping platforms could overcome this challenge (Crowell *et al.* 2016).

For sorghum breeding programs globally, obtaining locally-adaptive inflorescence morphology is essential. In field-based phenotypic selection, inflorescence morphology is directly observable prior to pollination. However, a shift to rapid-cycling genomics-enabled breeding in controlled conditions (Watson *et al.* 2018) would require accurate marker selection or genome prediction of inflorescence morphology along with other agronomic traits. Since the NAM founders originated from diverse agroclimatic zones, the genotype-phenotype map we developed should be relevant for sorghum breeding and genetics programs globally.
